# *SOAT1* missense variant in two cats with sebaceous gland dysplasia

**DOI:** 10.1007/s00438-023-02020-6

**Published:** 2023-04-15

**Authors:** Sarah Kiener, Barbara G. McMahill, Verena K. Affolter, Monika Welle, Julie A. Yager, Vidhya Jagannathan, Tosso Leeb

**Affiliations:** 1 Institute of Genetics, Vetsuisse Faculty, University of Bern, Bremgartenstrasse 109a, 3001 Bern, Switzerland; 2 Dermfocus, University of Bern, 3001 Bern, Switzerland; 3 Pathology Services, IDEXX Reference Laboratories Inc., Lander, WY 82520 USA; 4 Department of Pathology, Microbiology, Immunology, School of Veterinary Medicine, University California Davis, Davis, CA 95616 USA; 5 Institute of Animal Pathology, Vetsuisse Faculty, University of Bern, 3001 Bern, Switzerland; 6 Department of Pathobiology, Ontario Veterinary College, University of Guelph, Guelph, ON N1G 2W1 Canada; 7 School of Veterinary Science, University of Sydney, Sydney, NSW 2006 Australia

**Keywords:** *Felis catus*, Cat, Dermatology, Genodermatosis, Animal model, Precision medicine, Veterinary medicine

## Abstract

Spontaneously arisen hereditary diseases in domestic animals provide an excellent opportunity to study the physiological functions of the altered genes. We investigated two 4-month-old sibling domestic short haired kittens with dry dark debris around the eyes, nose, and ears, dark crusting on the legs and a thin poor hair coat. Skin biopsies revealed abnormal sebaceous gland morphology with lack of normal sebocyte arrangement and differentiation. Hair follicles had a distorted silhouette, interpreted as a change secondary to the observed sebaceous gland dysplasia. Whole genome sequencing on both affected kittens and 65 genetically diverse feline genomes was performed. Filtering for variants that were present in both kittens but absent from the control genomes revealed a homozygous missense variant in *SOAT1*, encoding sterol O-acyltransferase 1. The protein is localized in the endoplasmic reticulum and catalyzes the formation of cholesteryl esters, an essential component of sebum and meibum. The identified *SOAT1*:c.1531G > A variant is predicted to change a highly conserved glycine residue within the last transmembrane domain of SOAT1, p.Gly511Arg. In mice, variants in *Soat1* or complete knockout of the gene lead to the “hair interior defect” (hid) or abnormal Meibomian glands, respectively. *SOAT1*:c.1531G > A represents a plausible candidate variant for the observed sebaceous gland dysplasia in both kittens of this study. The variant was not present in 10 additional cats with a similar clinical and histopathological phenotype suggesting genetic heterogeneity. *SOAT1* variants should be considered as potential cause in hereditary sebaceous gland dysplasias of humans and domestic animals.

**Supplementary Information:**

The online version contains supplementary material available at 10.1007/s00438-023-02020-6.

## Introduction

Sebaceous glands are small exocrine glands and produce sebum, which is a complex mixture of lipids. Sebum composition differs between species, most likely due to species-specific functional requirements (Picardo et al. 2009). Most sebaceous glands are associated with hair follicles where they constitute a crucial component of the pilosebaceous unit. Sebum is released by holocrine secretion into the follicular canal (Montagna 1967; Geueke and Niemann 2021). The secretion promotes skin barrier function, contributes to proper hair follicle growth and homeostasis, serves as hydrophobic shield for the hair coat, and plays a dynamic role in thermoregulation (Zouboulis 2010; Shamloul and Khachemoune 2021a; Zouboulis et al. 2022). Sebum fulfills additional functions such as eccrine emulsification, synthesis of cytokines, chemokines, interleukins, pheromone and fatty acids, acid mantle formation, and hormone production (Shamloul and Khachemoune 2021a). Glands are larger and more numerous on the face, external auditory canal, and anogenital surfaces.

Meibomian glands are modified sebaceous glands on the palpebral border, which secrete meibum into the tear fluid to prevent its evaporation and protect the ocular surface (Montagna 1967; Shamloul and Khachemoune 2021a). Meibum has a unique composition of neutral lipids different from sebum (Butovich 2017).

Abnormal sebaceous gland activity has been implicated in a number of medical conditions and defective sebaceous glands have been linked to a variety of skin disorders (Shamloul and Khachemoune 2021b; Geueke and Niemann 2021).

Numerous mouse mutants with abnormal sebaceous glands have been reported (Ehrmann and Schneider 2016). However, it often remains unclear whether the observed phenotypes are a direct consequence of aberrant sebaceous gland development and/or sebaceous gland activity or rather unspecific secondary changes of other more general skin and hair follicle defects (Geueke and Niemann 2021).

In cats, reports about primary sebaceous gland disorders are rare (Scott 1989; de Sepibus et al. 2004). Sebaceous adenitis in cats is usually associated with mural folliculitis and either of unknown cause or associated with an internal malignancy (Scott et al. 1995; Pascal-Tenorio et al. 1997; Gross et al. 2001; Rottenberg et al. 2004; Singh et al. 2010; Linek et al. 2015; Kasabali et al. 2017).

Yager et al*.* reported ten kittens with a congenital dermatosis and abnormal sebaceous gland morphology (Yager et al. 2012). To the best of our knowledge, so far, no causal genetic variants for sebaceous gland related pathologies in domestic animals have been reported in the scientific literature.

The aim of the present study was to characterize the clinical and histopathological features of two cat siblings with striking skin abnormalities and to investigate a possible underlying genetic defect.

## Materials and methods

### Ethics statement

The cats in this study were privately owned and skin biopsies and blood samples for diagnostic purposes were collected with the consent of their owners. The collection of blood samples was approved by the Cantonal Committee for Animal Experiments (Canton of Bern; permit BE71/19). All animal experiments were done in accordance with local laws and regulations.

### Clinical and histopathological examinations

A physical examination of the two index cases was performed by the attending veterinarians. Two to four 4–6 mm skin punch biopsies per cat were taken and routinely processed for histopathology. Hematoxylin and eosin (H&E) stained slides were reviewed by board certified veterinary pathologists (B.G.M., V.K.A., J.A.Y.). A full necropsy was performed after euthanasia.

### Animal selection for genetic analyses

The study included a total of 728 cats. Two of them represent the index cases for the sebaceous gland dysplasia phenotype described in this study. During the course of this study, we investigated samples from ten additional cats with similar clinical and histopathological phenotypes. Information on all 12 cases diagnosed with sebaceous gland dysplasia is compiled in Table S1. The remaining 716 cats represent a genetically diverse convenience cohort from the Vetsuisse Biobank. No consistent phenotype information on these cats is available and they were considered population controls.

### DNA extraction

Genomic DNA was extracted from EDTA blood, native tissue samples, or formalin-fixed paraffin-embedded (FFPE) tissue samples. Extractions were performed with the Maxwell® RSC Whole Blood DNA Kit, Maxwell^®^ RSC PureFood GMO and Authentication Kit, or Maxwell^®^ RSC DNA FFPE Kit, respectively, on a Maxwell^®^ RSC 48 instrument (Promega, Dübendorf, Switzerland).

### Whole genome sequencing and variant calling

The genome of both affected kittens was sequenced at 20 × coverage using PCR free libraries on an Illumina NovaSeq 6000 instrument. The sequencing data was mapped to the genome reference and variant calling was done as described before (Jagannathan et al. 2019; Kiener et al. 2021). Here, we used the cat genome reference assembly F.catus_Fca126_mat1.0 and NCBI Annotation Release 105 (https://www.ncbi.nlm.nih.gov/genome/annotation_euk/Felis_catus/105/). For the filtering of private variants, we used previously obtained genome sequences from 65 genetically diverse cats (Table S2). All sequence data were deposited at the European Nucleotide Archive and accession numbers are listed in Table S2.

### Targeted genotyping

We used Sanger sequencing to genotype the candidate variant (XM_011291017.4:c.1531G > A). PCR amplification, subsequent sequencing and data analysis was performed as described (Kiener et al. 2021). The primer sequences used for this experiment are given in Table S3.

## Results

### Clinical history

**Fig. 1:**
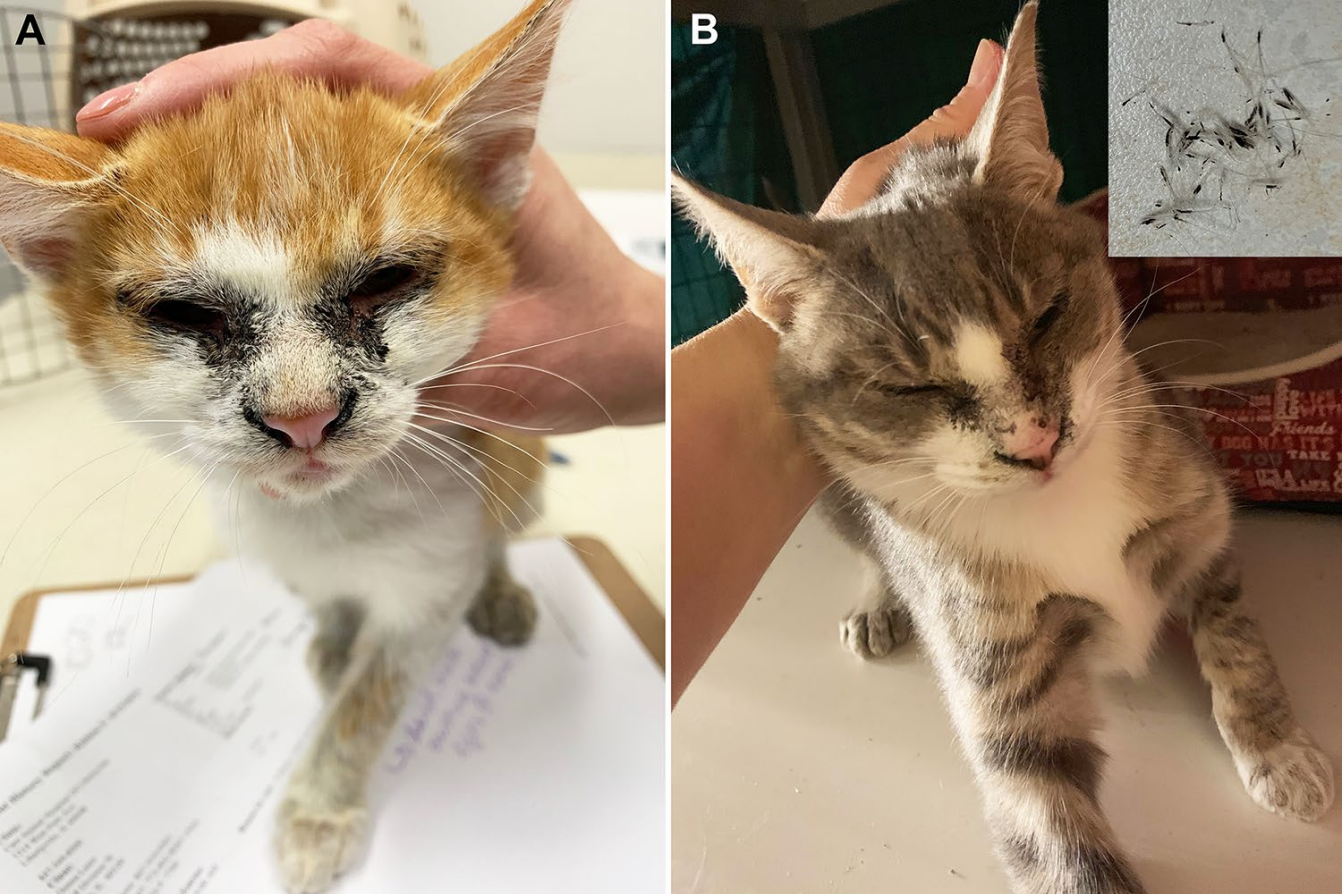
Clinical phenotype of affected kittens. **A** Case 2, bilateral symmetrical scaling with black debris, hair clumping and hypotrichosis periocular and surrounding the nostrils and legs. **B** Case 1, similar changes to case 2, however, less florid. Insert top right: note easily epilated clump of hairs with scaling and black debris

Five kittens born from a feral mother were presented at a shelter for first examination at approximately 4 months of age. Two kittens, one male and one female, had similar skin lesions of dry dark brown to black debris around ears, eyes, nares and dark crusting along legs associated with partial alopecia (Fig. 1A). The remaining hair coat was thin, in poor condition, and easily epilated. Other skin surface areas were covered with the same brown material to a lesser degree. The lesions persisted, progressed and failed to completely resolve over a time period of 4 months despite multiple attempted treatments (shampoo bathing, antibiotics, food trial, olive oil bathing, Revolution^®^, Convenia^®^, terramycin, terbinafine, famciclovir). Shampoo bathing and famciclovir seemed to have helped the most, but still resulted in very little improvement. Concurrent dermatophytosis (*M. canis*) developed and was treated when skin biopsy was elected. Both affected kittens were euthanized due to persistent skin problems and unlikelihood of adoption. The mother and the other littermates did not have skin lesions.

### Histopathological examination

**Fig. 2:**
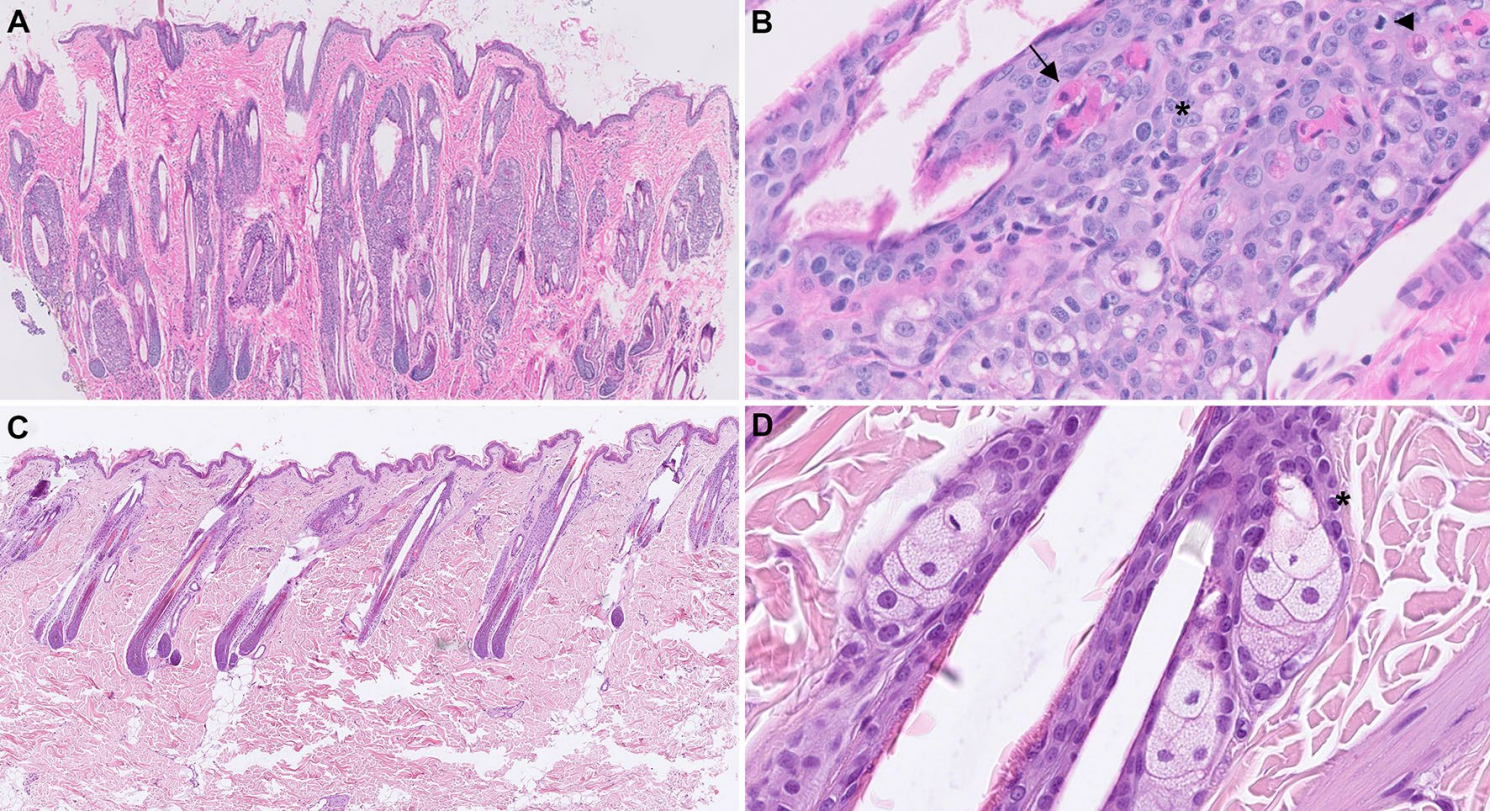
Histopathological phenotype of an affected kitten (case 2). **A** Case 2, variably prominent sebaceous glands with abnormal morphology, follicular dysplasia and basket weave hyperkeratosis, H&E 20x. **B** Case 2, abnormal sebaceous gland arrangement and differentiation characterized by increased number of reserve cells (asterisk), apoptotic cells (arrow) and mitotic activity (arrowhead), H&E 400x. **C** Age matched control, normal pilosebaceous morphology with well differentiated sebaceous glands at the isthmus level, H&E 20x. **D** Normal sebaceous gland maturation from reserve cell (asterisk) to mature sebocytes, H&E 400x

The main pattern observed was the generalized abnormal sebaceous gland morphology identified in both primary skin biopsies and post mortem samples of skin (Fig. 2A, B). Some glands were proliferative with reserve cell predominance, increased mitotic activity and apoptosis (Fig. 2B). The apoptosis was not associated with satellitosis. There was an almost total absence of normal differentiation from reserve cells to mature lipid laden sebocytes. The number of mature sebocytes was decreased. Peri-isthmus lymphocytic infiltrates were present around some sebaceous glands with minimal to absent follicular mural involvement. Most hair follicles were in anagen phase. There was evidence of follicular dysplasia with wavy contours and few misshapen anagen bulbs. Dark brown to black discoloration of follicular keratin was visible, as well as frequent misshapen and malacic hair shafts, some of which were mineralized. Superficial perivascular inflammation was very minimal to absent and likely secondary to superficial infections. The epidermis showed moderate to marked orthokeratotic hyperkeratosis and was variably acanthotic. These findings led to a final diagnosis of diffuse sebaceous gland dysplasia with follicular dysplasia, hair shaft malacia, and moderate to marked hyperkeratosis.

### Genetic analysis

We performed whole genome sequencing on both affected kittens and compared the data to 65 genomes from genetically diverse cats. Hierarchical filtering steps were applied to identify a candidate causative variant for the sebaceous gland dysplasia phenotype. Of all variants present in both kittens, we filtered for shared private variants, i.e. variants that were present in both kittens but absent in the 65 control genomes that we used. We subsequently filtered these for protein-changing variants with a SnpEff impact of “high” or “moderate.” The final step prioritized the variants for functional candidate genes. These filtering steps identified a clear top candidate variant that can be designated as ChrF1:20,914,140G > A with respect to the F.catus_Fca126_mat1.0 genome reference assembly (Table 1, Tables S2, S4).

**Table 1:** Summary of the variant filtering steps in both kittens affected with sebaceous gland dysplasia

Filtering step	Homozygous	Heterozygous
Shared variants in both affected kittens	3,944,823	3,181,925
Shared private variants in both affected kittens	4006	34,891
Shared private and protein-changing variants	16	157
In plausible functional candidate genes	1	0

**Fig. 3:**
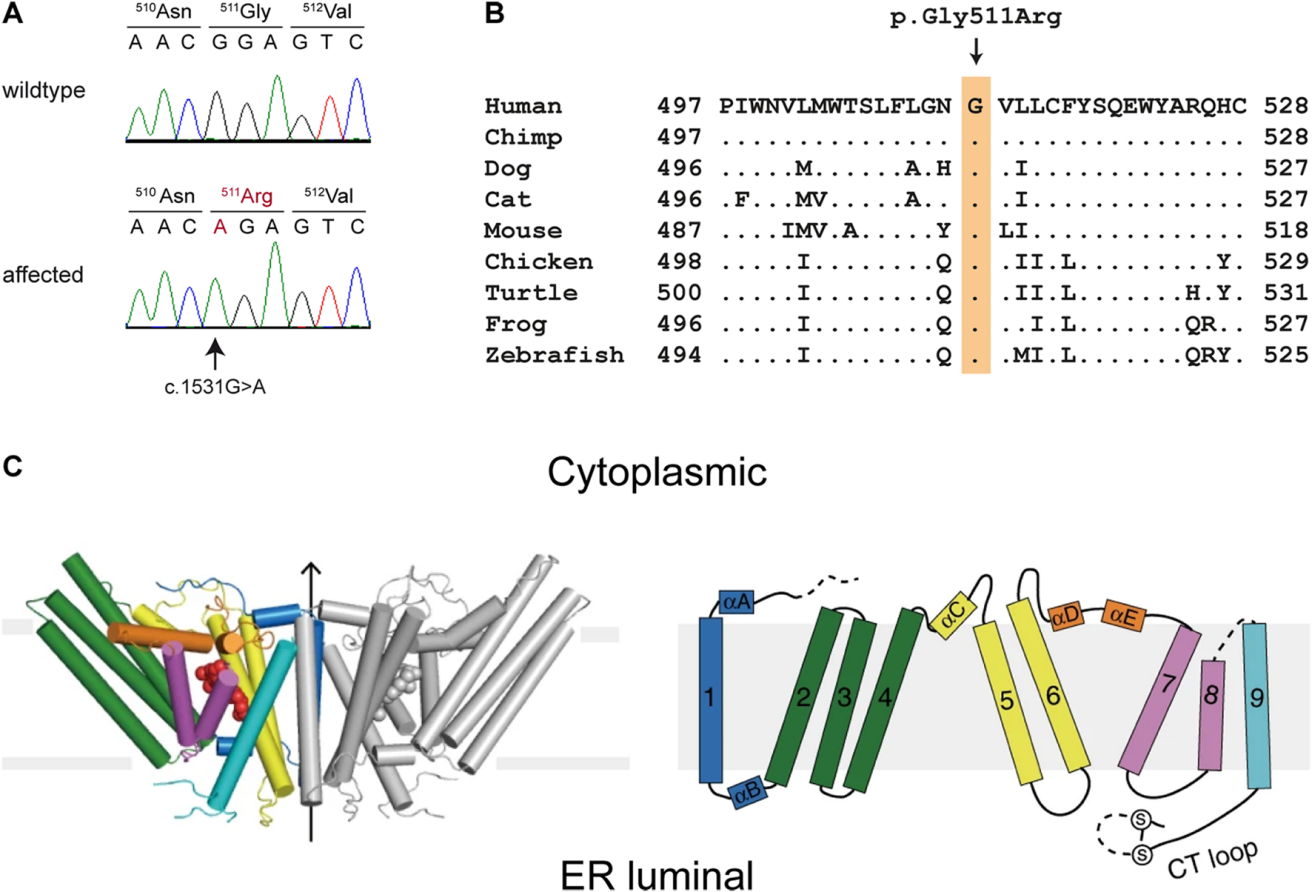
Details of the *SOAT1*: c.1531G > A, p.Gly511Arg variant. **A** Electropherograms with the amino acid translations of two cats with different genotypes. The arrow indicates the single nucleotide change. **B** Multi-species sequence alignment of the last transmembrane domain of SOAT1 harboring the p.Gly511Arg variant. **C** Structural model of a SOAT1 dimer in side view (left image) and the topological model of SOAT1 (right image). The full functional enzyme consists of a dimer of dimers (images from Guan et al. 2020)

The identified variant represented a missense variant in the *SOAT1* gene, XM_011291017.4:c.1531G > A. It is predicted to change an evolutionarily conserved glycine residue in the last transmembrane domain of sterol O-acyltransferase 1, XP_011289319.1:p.(Gly511Arg) (Fig. 3). The effect of the amino acid exchange was classified as deleterious or pathogenic by the variant impact predictors PredictSNP (87% probability, Bendl et al. 2014), Provean (score − 6.610, Choi and Chan 2015), and MutPred2 (score 0.767, Pejaver et al. 2020).

We confirmed the presence of the c.1531G > A variant in genomic DNA of both affected kittens by Sanger sequencing. Both kittens were homozygous for the mutant allele, consistent with an autosomal recessive mode of inheritance. We further genotyped archived samples from 10 additional cats with a similar clinical and histopathological phenotype, as well as 716 genetically diverse population controls from the Vetsuisse Biobank, all of which were homozygous wildtype (Table 2).

**Table 2:** Genotype–phenotype association at the *SOAT1*:c.1531G > A variant with sebaceous gland dysplasia

Cats	G/G	G/A	A/A
Affected kittens (littermates), index cases (*n* = 2)	–	–	2
Unrelated affected cats (*n* = 10)	10	–	–
Unrelated control cats (*n* = 716)	716	–	–

## Discussion

The two kittens investigated in this study displayed a distinct phenotype, which we refer to as sebaceous gland dysplasia. This unique constellation of clinical and histopathological changes was initially described in ten unrelated kittens (Yager et al. 2012). In accordance with this initial study, lesions developed at a very young age pointing towards a congenital disorder. Interestingly, a clinical presentation of progressive hypotrichosis/alopecia rather than scale was the predominant feature in the previously reported kittens (Yager et al. 2012). In addition to hypotrichosis/alopecia, the two index cases of the present study had a bold black debris covering the skin surface, which we interpreted as adherent oxidized sebum and keratin. Histopathological findings were unique with diffusely abnormal sebaceous gland morphology. The characteristic histopathology included disrupted sebaceous gland maturation with reduction of mature sebocytes, increased number of undifferentiated reserve cells and presence of apoptotic cells. In contrast to the ten cases reported previously (Yager et al. 2012), sebaceous glands looked proliferative in some areas and vacuolated sebocytes with prominent eosinophilic globules were not as obvious.

The pedigree with two out of five littermates being affected, the early onset and the phenotypic characteristics suggested a genetic cause. We﻿ therefore, investigated the genomes of the affected kittens and searched for plausible candidate variants. The whole-genome sequencing data of both kittens identified 173 protein-changing variants that were exclusively present and shared in these 2 cats, but absent from 65 control genomes. We prioritized genes based on the clinical and histologic findings, and one variant was of particular interest. It was a homozygous missense variant in *SOAT1*, encoding sterol O-acyltransferase 1. This intracellular protein is localized in the membrane of the endoplasmic reticulum, where it catalyzes the formation of cholesteryl esters from cholesterol and long chain fatty acyl-CoA. It thus maintains the ratio of free cholesterol and cholesteryl esters in the cells (Wu et al. 2010). Cholesteryl esters are stored in lipid droplets within the cell or transported to other tissues (Guan et al. 2020). They are important components of sebum and meibum and fulfill essential functions in the lipid envelope of the epidermis (Butovich 2017).

The mature SOAT1 protein contains nine transmembrane domains and is incorporated into the ER membrane as a dimer of dimers. The central cavity is formed by the six transmembrane helices 4–9, and the transmembrane helices 1, 6 and 9 are involved in dimer assembly (Guan et al. 2020; Long et al. 2020). The identified variant in the affected kittens from this study is predicted to change a highly conserved glycine residue in the ninth and last transmembrane domain, p.Gly511Arg. We hypothesize that this compromises the enzymatic activity.

In a genetically engineered *Soat1*-null mouse model (Meiner et al. 1996), the predominant phenotype is Meibomian gland dysfunction. *Soat*^*−/−*^ mice had notably smaller eye openings and very thick meibum with lipid-like debris around the eye openings. The Meibomian lipids showed cholesterol instead of cholesteryl esters as the dominant lipid. Abnormalities in their skin and fur coat were not visible (Butovich et al. 2021). However, the spontaneous "hair interior defect" (hid) caused by a 6.8 kb deletion in *Soat1* was reported in the AKR/J mouse (Trigg 1972; Wu et al. 2010). Characteristics of the hid-phenotype included hair with deficiency in projections of cortex cells and low levels of trichohyalin (Wu et al. 2010).

In the affected kittens from this study, the Meibomian glands were not examined. However, on clinical pictures, the eye openings were very small, possibly due to ocular surface irritation, similar to those of *Soat1*-null mice (Butovich et al. 2021). The results of both mouse models support our hypothesis that the identified feline *SOAT1* variant might cause the observed phenotype through a direct impact of defective SOAT1 on sebaceous gland development and/or function. Alternatively, an additional indirect effect of this variant has to be considered, as in vitro studies of *SOAT1*-knockdown cells showed a reduced expression of cholesterol metabolism genes and fatty acid biosynthesis genes (Zhu et al. 2021). Deficiency of *Scd1* encoding stearoyl-coenzyme A desaturase 1 also results in aberrant sebaceous and/or Meibomian glands in mice (Ehrmann and Schneider 2016).

Unfortunately, both kittens from our study were euthanized shortly after presentation and before the genetic investigations were completed. Therefore, no suitable samples for functional follow-up such as an analysis of the lipid content of the skin and tear fluid were available.

Since the clinical and histopathological features of the cat siblings from this study were similar to those described by Yager et al. (2012), we genotyped ten additional cases with similar phenotypic changes including one cat from the Yager et al. (2012) study for the identified variant. All ten additional cats were homozygous for the wildtype allele at the *SOAT1*:c.1531G > A variant. As already previously proposed, the term sebaceous gland dysplasia does not represent a homogeneous condition, but rather the phenotypic expression of more than one disorder of sebaceous gland development (Yager et al. 2012). Our genotyping results support this statement and suggest genetic heterogeneity of this phenotype.

In conclusion, we describe the clinical and histopathological findings of two kittens with sebaceous gland dysplasia. Whole-genome sequencing revealed a homozygous candidate variant in *SOAT1*, p.Gly511Arg, as a potential and highly plausible underlying defect. Further studies are required to evaluate the exact functional impact of the variant. Our results propose a new candidate gene for sebaceous gland dysplasia phenotypes, which might be relevant for future unsolved cases in veterinary and human medicine.

## Supplementary Information

Below is the link to the electronic supplementary material.

Supplementary file1 (XLSX 13 KB)

Supplementary file2 (XLSX 13 KB)

Supplementary file3 (XLSX 10 KB)

Supplementary file4 (XLSX 9824 KB)

## Data Availability

The genome sequence data were submitted to the European Nucleotide Archive (ENA). All accession numbers are listed in Table S2.
